# Camel Calves as Opportunistic Milk Thefts? The First Description of Allosuckling in Domestic Bactrian Camel (*Camelus bactrianus*)

**DOI:** 10.1371/journal.pone.0053052

**Published:** 2013-01-09

**Authors:** Karolína Brandlová, Luděk Bartoš, Tamara Haberová

**Affiliations:** 1 Department of Animal Science and Food Processing in Tropics and Subtropics, Institute of Tropics and Subtropics, Czech University of Life Sciences Prague, Praha, Czech Republic; 2 Department of Ethology, Institute of Animal Science, Praha Uhříněves, Czech Republic; 3 Department of Animal Science and Ethology, Faculty of Agrobiology, Food, and Natural Resources, Czech University of Life Sciences Prague, Praha, Czech Republic; University of Rennes 1, France

## Abstract

Allosuckling is a situation when a female nurses a non-filial offspring. It was described in various ungulate species; however for camels this is the first description of this behaviour. The aim of the study was to assess the occurrence of allosuckling in captive camels (*Camelus bactrianus*) and to test whether it can be explained as a ‘milk-theft’ (opportunistic behaviour of calves) or alternatively as an altruistic behaviour of females. During 2005 and 2007, nine camel females and ten calves in four zoological gardens in the Czech Republic were observed. In total, 373 sucking bouts were recorded, from which 32 were non-filial (the calf sucked from the non-maternal female). Allosuckling regularly appeared in captive camel herds. As predicted for the milk-theft explanation, the non-filial calves sucked more often in the lateral position and even did not suck in the antiparallel position at all. The non-filial calves preferably joined the filial calf when sucking but in five cases (15.6% of non-filial sucking bouts) the calves sucked from non-maternal dam without the presence of filial calf. We then expected the differences in terminations of sucking bouts by females but did not find any difference in sucking terminations for filial and non-filial calves. As the calves were getting older, the incidence of allosucking increased. This was probably because skills of the calf to outwit the non-maternal dam increased and/or the older calves might be more motivated for allosucking due to the weaning process. Finally, duration of a sucking bout was shorter with non-filial than filial calves. The results of the study support the hypothesis of ‘milk theft’, being mostly performed by calves behaving as opportunistic parasites, but we cannot reject certain level of altruism from the allonursing females or their increased degree of tolerance to non-filial calves.

## Introduction

Allonursing or communal nursing, communal suckling, non-offspring nursing in mammals refers to the situation when a lactating female nurses a young which is not her own [1], [2]. When an offspring sucks milk from female which is not its mother, we call this allosucking or communal sucking [3], [4]. We use the term allosuckling for both behaviours together. This phenomenon can be explained as an extreme form of communal maternal care [5], [6], known in various mammalian orders [1]. Nevertheless, the explanations of allosuckling occurrence are diverse across the species and situations and functions of allonursing are not well understood. Allosuckling involves tolerance by nursing females, ranging from kin directed discrimination or social affiliation of females [2], [7], [8], to a parasitic behaviour of young in which they steal milk without the female's acceptance [1], [2]. Motivation of calves for allosuckling is often explained as a compensation of nutritional requirements of the young [9], [10], using either the tolerance of females or a milk-theft strategy [9]–[13]. The explanation of allosuckling as an adaptive behaviour of females involves mostly the kin selection hypothesis [1], [2], [4], [10] in which females nurse preferably the offspring of related females, or the reciprocity hypothesis, when females nurse the offspring of another group member reciprocally [14], [15]. The reciprocity hypothesis in general is expected to apply in stable groups of social animals and is therefore connected with social affiliation [8]. In accordance with the compensation theory, females in better body condition may be more tolerant to non-filial calves. In some extreme cases a female actively nurses a non-filial offspring because she does not recognize that the offspring is not her own [16]. Roulin [2] calls this behaviour the misdirected parental care, connecting it with milk-theft. The milk-theft hypothesis [1] predicts that the calf tries to ‘steal’ the milk from a non-maternal female, but when the female recognizes the calf is not her own, she would refuse to nurse it as observed e.g. in various seal species [17]. This behaviour is found more in overcrowded conditions and is more frequent in captive populations [1], [2], [18].

The dromedary camel (*Camelus dromedarius*) is mentioned in the list of mammals published by Packer *et al.* [1] as the species with no allosuckling occurrence. However, allosuckling has been described in other camelids. Zapata *et al.* [19] reported incidental allosuckling occurrence in wild guanacos (*Lama guanicoe*) and regular occurrence of allosuckling in captive farmed guanacos as behaviours that were consistent with the milk-theft hypothesis and a compensation theory [20], [21].

The wild Bactrian camel (*Camelus ferus*) is now considered like a separate species [22] and is found exclusively in China and Mongolia [23]. No information about the suckling behaviour of wild camels has been published. The Bactrian camels kept in European breeding facilities belong to the domestic form (*Camelus bactrianus*) [24]. Camels are monotocous ungulate species, having only one offspring per litter [25], [26]. A female in feral or extensively bred camels usually leaves the herd for parturition, while in captivity is often separated from the herd by keepers. The calf follows the mother for several hours after birth. Within a week the mother and the calf rejoin the herd [27], [28]. According to our observations in zoo camels, females which are not separated often give birth surrounded by the other herd members. Camels are seasonal breeders and the calves are born during spring months [25]. In European breeding facilities the breeding season is prolonged and calves are born all over the year with a peak in spring months [24]. Camel calves are nursed up to two years and the female can have a calf every two years [28]. Some of the females may give birth every year [29]. According to the study of Sambraus [30], camel calves sucked 8 times in 24 h period, slighter more during the daylight. Nursing dams did not limit sucking of calves up to 3 months of age, while they frequently terminated sucking bouts of older calves [30].

The aim of this study was to provide the first description of allosuckling occurrence in camels and to test possible hypotheses explaining this behaviour. The kin selection hypothesis did not seem to be a major factor in this study, as the females were not related to each another. Based on the findings of Zapata *et al.* [19], [20] on another camelid, the guanaco, we predicted the milk-theft hypothesis be the main cause why the camel calves sucked from non-maternal females. If this was valid we predicted that (i) a calf would suck from the non-maternal dam standing in other than antiparallel sucking position so that it was more difficult for the dam to distinguish the calf's identity or to threat the non-filial calf. (ii) A calf should preferably join the filial calf during sucking non-maternal dam. (iii) If the position served as a tactic not to be recognized or threatened, one would expect termination of a sucking bout involving a non-filial calf in an antiparallel position (if any) by the dam be more frequent than if the non-filial calf was sucking in a lateral position. We also predicted that (iv) the incidence of allosucking will increase with age of the allosucking calf as skills of the calf to outwit the non-maternal dam would increase or the calf will be more motivated for allosuckling. Finally, we predicted (v) duration of a sucking bout will take shorter time with non-filial than filial calves. Alternatively, if the result will not correspond with the milk-theft hypothesis, an altruistic behaviour of females should be taken in account, either in the form of reciprocal help or compensation.

## Materials and Methods

### Ethic statement

Observations of camels were carried out in zoos mostly from the visitors' area or from the background yards when needed. The observer did not enter animal enclosure and did not affect the behaviour, husbandry, and management of studied animals. The zoo managers were informed and agreed with the research activities.

### Animals and husbandry

From 2005 to 2007, we have studied maternal behaviour of Bactrian camels kept in four zoological gardens in the Czech Republic (Praha, Brno, Ostrava, Zlín–Lešná). Nine females (one of them reproduced two times within the observation period) and ten calves (4 males, 6 females), were included in the study. The size of herds ranged between 5 and 11 individuals; including 2 to 3 calves (Table 1). All calves in each herd were sired by the same bull, making them half-siblings to one another. Females were not related to one another, but have lived together most of their lives. All except one female were multiparous. Additional data on calves are presented in Table 2. Each animal was identified individually, according to the shape of humps, hair and facial traits. Age, origin, kinship, and other attributes of females were available according to Animal Record Keeping System (ARKS) records of every zoo (see Table 3 for details).

**Table 1:** Zoological gardens included in the study with the numbers of camels kept and the number of filial and non-filial sucking bouts in herds.

Zoo	Year	Adults (M, F)	Nursing F	Calves (M, F)	Total SB	Non-filial SB	Non-filial SB (%)
**Brno**	**2006**	**1,4**	**2**	**0,2**	**81**	**0**	**0,00**
**Brno**	**2007**	**1,3**	**1**	**0,2** *	**26**	**3**	**11,54**
**Zlín-Lešná**	**2005**	**1,2**	**2**	**2,0**	**58**	**2**	**3,45**
**Ostrava**	**2006**	**1,7**	**2**	**1,1**	**85**	**16**	**18,82**
**Ostrava**	**2007**	**1,7**	**3**	**1,2**	**36**	**9**	**25,00**
**Praha**	**2006**	**1,5**	**2**	**1,1**	**87**	**2**	**2,29**
Total					373	32	**8,58**

(M - males; F- females; SB – sucking bout).

* One of the calves was already weaned by its mother but occasionally sucked from a non-maternal dam.

**Table 2:** Camel calves included in the study with the number of observed hours.

Zoo	Year	Name	Date of birth	Sex	Mother	Age of calf (months)	Observed hours	No. of sucking bouts	Allo-sucking extent (%)	Allo-sucker	Mean duration ± SE of filial sucking (sec.)	Mean duration ± SE of allosucking (sec.)
Brno	2006	April	2.4.2006	F	Isis	2–5	32	75	0,00	NO	22.48±2.38	
Brno	2006	Gaja	18.4.2005	F	Sulika	13–16	32	6	0,00	NO	50.50±22.41	
Brno	2007	April	2.4.2006	F	Isis	14–17	24	3	100,00	YES		37.33±16.83
Brno	2007	Polednice	10.3.2007	F	Sulika	2–5	24	23	0,00	NO	33.87±6.58	
Zlín-Lešná	2005	Marek	17.5.2005	M	Jade	0–3	22	34	0,00	NO	55.15±10.41	
Zlín-Lešná	2005	Aštar	14.3.2005	M	Klaudie	3–6	22	24	8,33	YES	75.91±15.66	10.00±0.00
Ostrava	2006	2sameček	31.3.2006	M	Vendula	2–6	42,5	30	3,33	YES	62.90±9.03	5.00±0.00
Ostrava	2006	1samička	23.3.2006	F	Čora	2–6	42,5	55	27,27	YES	41.23±5.36	36.80±7.39
Ostrava	2007	2sameček	31.3.2006	M	Vendula	14–16	16	8	50,0	YES	41.25±15.20	58.50±24.15
Ostrava	2007	1samička	23.3.2006	F	Čora	14–16	16	11	45,45	YES	32.50±7.06	43.80±14.45
Ostrava	2007	Kobi	2.3.2007	F	Fatima	2–4	16	17	0,00	NO	46.06±10.05	
Praha	2006	Vanda	18.1.2006	F	Lee	6–8	29	43	4,65	YES	37.15±4.96	17.50±5.50
Praha	2006	Víťa	21.7.2006	M	Rona	0–3	29	44	0,00	NO	54.16±6.63	

**Table 3:** Camel females included in the study.

Zoo	Female name	Birthdate	Arrival to present zoo	Parity till 2005	Number of calves till 2005	Parity till 2006	Number of calves till 2006	Parity till 2007	Number of calves till 2007	Allonursing extent (%)	Allonurser
Brno	Isis	2.6.1998	24.6.1999	2	1	3	2	3	2	0.00	NO
Brno	Sulika	6.3.1992	6.3.1992	7	5	7	5	8	6	9.38	YES
Zlín-Lešná	Jade	24.2.1997	3.9.1998	4	4	4	4	5	4	5.56	YES
Zlín-Lešná	Klaudie	3.5.1999	3.5.1999	2	1	2	1	3	2	0.00	NO
Ostrava	Vendula	03.04.2000	30.5.2001	1	0	2	1	2	1	35.29	YES
Ostrava	Čora	02.03.2002	30.5.2003	0	0	1	1	1	1	2.13	YES
Ostrava	Fatima	17.05.1990	27.6.2003	2	0	2	0	3	1	26.09	YES
Praha	Lee	02.06.1998	24.6.1999	2	1	3	2	4	3	0.00	NO
Praha	Rona	15.03.1995	26.4.1996	4	1	5	2	5	2	4.35	YES

Camels in all facilities were fed once or twice a day by hay and grasses *ad libitum*, supplemented with grains and vegetables, and *ad libitum* water supply. The animals were kept outdoors, mostly with the access to unheated stables or shelters. The outdoor enclosures of camels in the zoos had mostly grass or sandy surface with a similar space allowance in all cases. Even in larger enclosures camels spent most of the time close to each other and were not dispersed. The daily maintenance of herd was done by the keepers either entering the herd directly or moving animals from the stable to enclosure and back to clean all the space without the direct contact with animals. Females were separated before parturition and joined the rest of the herd after two to 30 days of the calves' life.

### Recorded variables

We recorded all occurrences of suckling by *ad libitum* sampling method [31]. Selected activities were directly observed by one observer (Karolína Brandlová). The observations were performed monthly in all studied calves during 7–10 hours a day (0800–1800, 0800–1700, 0900–1800, 0800–1600), depending on locality and season, starting as soon as possible after birth of the second calf in the respective herd and continuing at least 3 months.

For each sucking bout we recorded the identity of the animals, duration of sucking bout, position of sucking calf, which animal terminated the sucking bout (mother, calf, or other). The position of the sucking calf was classified into two classes - antiparallel, when the hind part of the calf was directed toward a cow's head, and lateral, when the calf stands at least in the right angle to the cow's body axis. As the gap between the start of sucking and milk let-down is not documented in camels, we consider all bouts longer than 5 seconds as successful as in other studied species e.g. [4], [32], [33]. Sucking bout was considered to terminate when it was interrupted for at least 10 seconds.

### Assessment and statistics

The data were analysed using Statistical Analysis Systems (SAS) version 9.2. Frequency counts for prediction (i) were analysed by computing chi-square test (PROC FREQ). The output contained cell or cells counts less than 5, hence Pearson exact chi-square was used. For other data we used Generalised Linear Mixed Model (GLMM) for analysing numeric variables (PROC MIXED) or categorical variables (PROC GLIMMIX for binary distribution). To account for repeated measures, all mixed model analyses but one were performed using individual camel ‘calf’ nested within the ‘herd’ as a random effect. In unbalanced designs with more than one effect, the arithmetic mean for a group may not accurately reflect response for that group, because it does not take other effects into account. Therefore, we used least-squares-means (LSMEANs) instead. LSMEANs are, in effect, within-group means appropriately adjusted for other effects in the model. LSMEANs were computed for each class and differences between classes were tested by t-test. For multiple comparisons we used the post hoc Tukey-Kramer adjustment.

We combined predictions (ii) and (iv) into one GLMM for binary distribution modelling the probability for a calf to suck from non-maternal dam. Fixed effects were ‘age of the calf’ (a continuous predictor that ranged from 1 to 17 months), ‘number of calves’ taking part in the sucking bout (a categorical factor with levels 1 to 3), ‘nursing females’ (a categorical factor with levels 2 and 3 females per herd),‘sex of the calf’ (a categorical factor with male and female levels), and ‘sucking order’ (a categorical factor with levels the 1st, the 2nd, and the 3^rd^ calf coming to suck). None of the non-filial calves sucked in antiparallel position, therefore the effect ‘Position’ (antiparallel or lateral) could not be applied. For prediction (iii), we applied a GLMM for binary distribution modelling the probability for a dam to terminate the sucking bout. Fixed effects were ‘relatedness’ (filial sucking and non-filial sucking), ‘position’, ‘age of the calf’, ‘sex of the calf’, ‘nursing females’, and ‘birth order’ (the birth order of the calf within the season and herd). Primarily we were interested in testing the effect of the ‘relatedness’ alone and/or in an interaction with ‘position’. Given that none of the non-filial calves sucked in the antiparallel position, the effect of ‘position’ had to be omitted. We also examined various combinations of the other fixed effects (i.e., ‘relation’, ‘age of the calf’, ‘sex of the calf’, ‘nursing females’, and ‘birth order’) on the termination of sucking by the dam. For prediction (v) we applied GLMM with duration of the sucking bout as a dependent variable. Fixed effects were ‘age of the calf’, ‘age of the dam’ (4 to 17 years), ‘number of calves’, ‘relatedness’ in interaction with ‘position’ and in interaction with ‘sex of the calf’.

## Results

Over the three years of study (2005–2007; 164 hours within 26 days of observation in total) we have recorded 373 sucking bouts (Table 1). The non-filial sucking represented 8.58% (32 out of 373) of all sucking bouts. In all herds, 50% of calves (5) sucked exclusively from their own mothers, and 50% sucked from both own mother and non-filial cows. Six out of nine (66.67%) cows nursed both filial and non-filial calves. Three cows nursed the filial calf exclusively (Table 3). In individual calves, allosucking ranged from 0 to 100% of all sucking bouts. For individual females, allonursing ranged from 0 to 35% of all nursing bouts. Calves allosucked from the females which had younger calves than the allosucking one. The youngest calf in the herd had never allosucked (Table 2).

### (i) Sucking position

**Figure 1:**
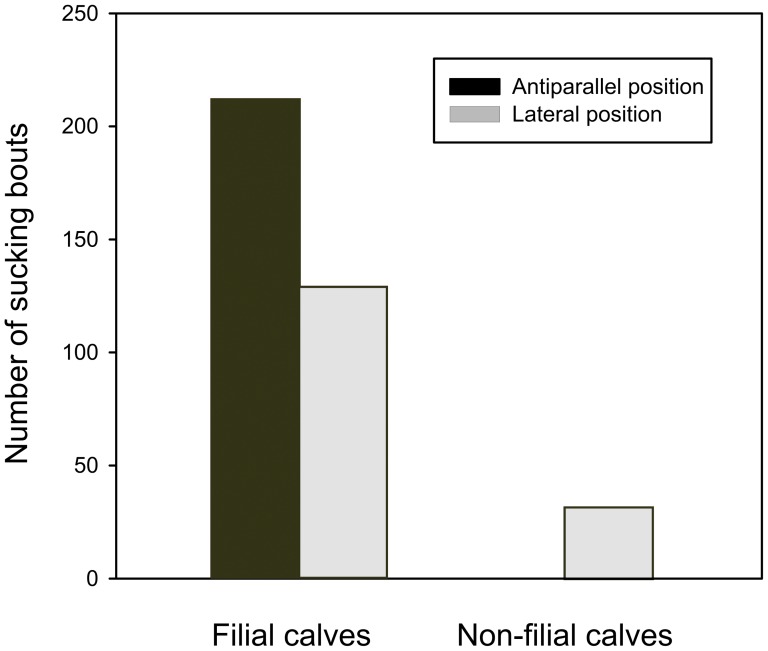
Sucking positions chosen by filial and non-filial camel calves.

Filial calves sucked from their mothers mostly standing in the antiparallel position (62.17% of cases), while non-filial calves suckled exclusively in the lateral position (n = 32, difference Pearson exact chi-square test p = 3.04 * 10^−13^, Figure 1).

### (ii) Number of sucking calves

**Figure 2:**
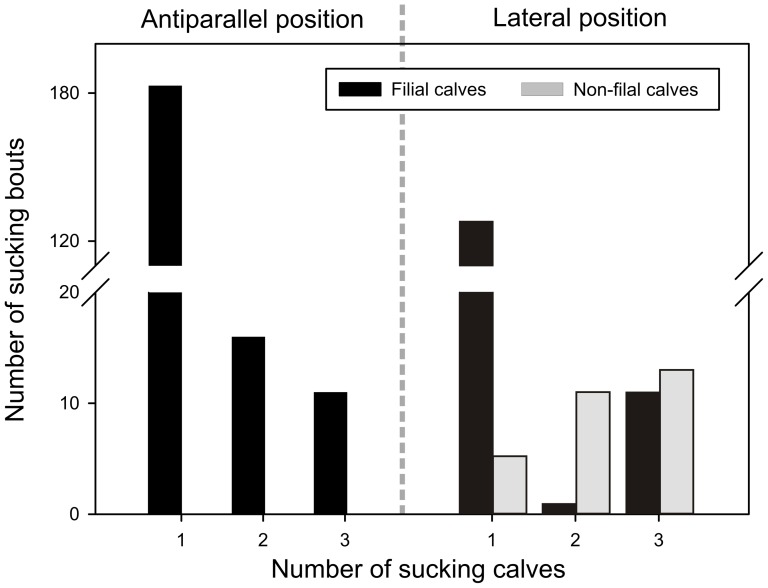
Suckling bout occurrence for filial and non-filial calves according to the number of sucking calves and position during sucking.

Four non-filial calves were involved in a sucking bout without the presence of filial calves five times (15.6% of cases), standing in a lateral position (Figure 2). In all 27 cases when non-filial calves were allosucking with other calf or calves present, they invariably joined already sucking filial calf.

### (iii) Termination of sucking by the dam

Termination of sucking by the dam was not affected by any of the tested factors either when they entered the model alone or in any combination with other factors. Non-filial calves never sucked in anti-parallel position, so we could not test the effect of position to termination. Of the non-filial calves which sucked without a presence of filial calf, sucking was terminated by the calf three times, once by the dam and in one case we did not see who terminated the bout.

### (iv) Sucking probability

**Figure 3:**
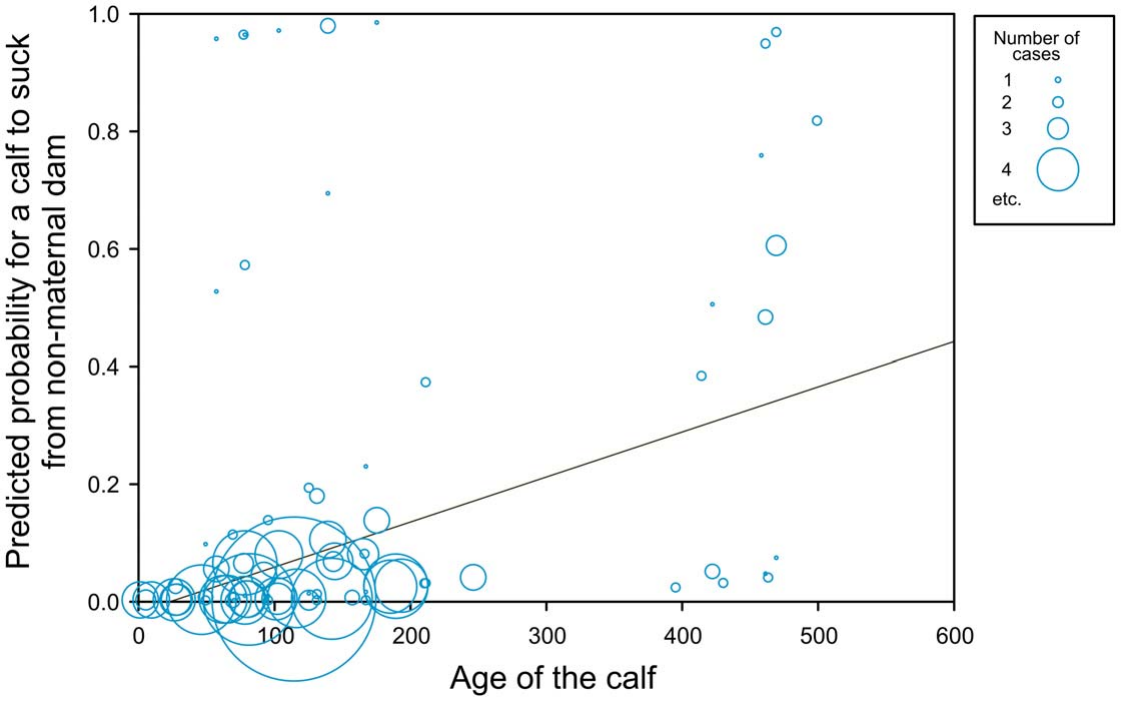
Probability of non-filial suckling bout occurrence according to calf age.

The GLMM model revealed that the probability for a calf to suck from non-maternal dam was affected by ‘age’ of the calf (F(1,358) = 3.96, p = 0.047, Figure 3), and ‘number of calves’ taking part in the sucking bout (F(1,358) = 27.50, p<0.0001). In particular, allosucking was more likely in older calves and with increasing number of sucking calves. ‘Nursing females’ and ‘sex of the calf’ were not significant predictors and were dropped from the model.

### (v) Sucking duration

The mean (± SE) sucking duration was 42.93±2.22 s (range 5–270), the mean duration of filial sucking bout 43.50±2.37 s (range 5–270) and the non-filial sucking bout 36.78±5.47 s (range 5–121).

**Figure 4:**
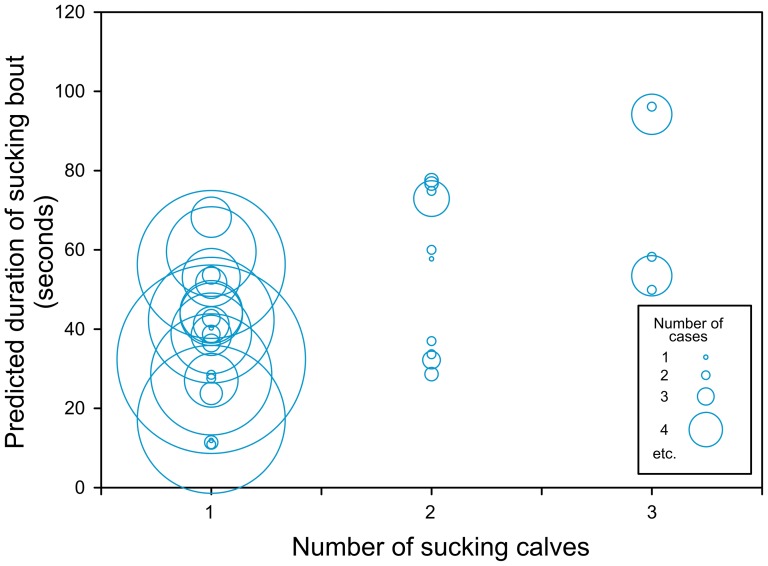
Sucking bout duration depended on number of calves.

**Figure 5:**
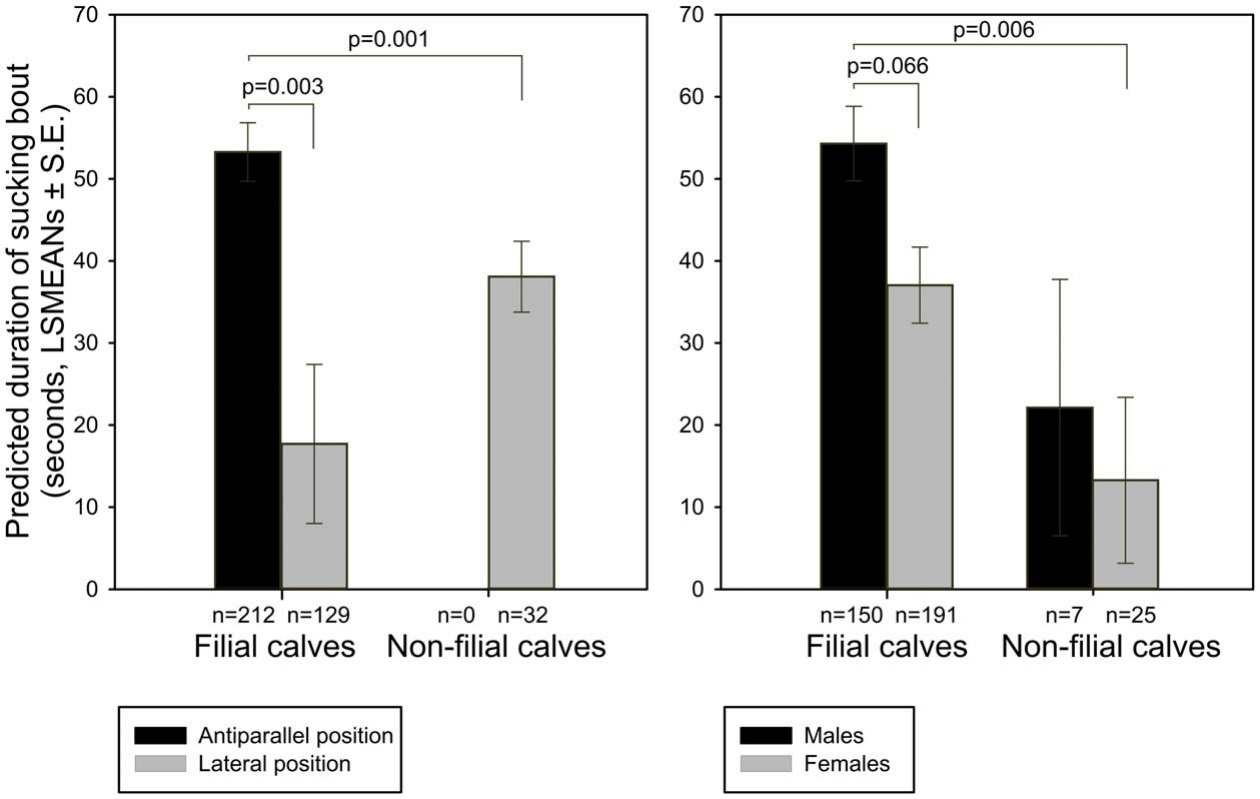
Sucking bout duration (LSMEAN ± SE) for filial and non-filial calves according to position and for filial and non-filial male and female calves.

The GLMM model showed that duration of sucking bouts was dependent on the ‘number of calves’ taking part in the sucking bout (F(1,191) = 17.19, p<0.0001), so the sucking bouts involving more than one calf were longer than those involving just one calf, either filial or non-filial (Figure 4). Duration of sucking bouts was also dependent on a relatedness by position interaction (F(1,367) = 11.05, p = 0.001), meaning that calves in the antiparallel position (only filial ones) sucked longer than those in the lateral position (either filial or non-filial) (Figure 5 left). Sucking duration depended also on a relatedness by sex interaction (F(2,20.7) = 3.49, p = 0.049), showing that the sucking bouts of filial males were longer than those of females, both filial and non-filial. Non-filial males did not differ from non-filial females (Figure 5 right). ‘Age of the calf’ and ‘age of the dam’ were not significant predictors and were removed from the final model. For non-filial calves only, duration of allosucking was much shorter when the non-filial calves were sucking alone (9±12.54 seconds) compared when there were one or two other calves (41.92±5.40 seconds, F(1,32) = 5.82, p = 0.02).

### Comparison between allonursing and non-allonursing dams

Comparison between allonursing and non-allonursing dams is shown in Table 3. The numbers of the animals are low for statistical comparison. Nevertheless, none of the characteristics available (age, parity, number of calves reared) seems to play any significant role in whether or not the dam allows non-filial calf to suck.

## Discussion

### Allosuckling in camels

In this study we brought the first description of allosuckling occurrence in camels. The results have shown that allosuckling occurred in 5 out of 10 calves from 4 camel herds containing more than one calf in different zoos and different seasons. The allosucking calves were in all cases the older ones in the herd, while the youngest calf from the herd never allosucked. The only herd in the study without allosucking occurrence was the Brno Zoo in 2006, where two female calves from different mothers were kept together. Although the data were not included in the study, one author (Karolína Brandlová) observed the allosuckling occurrence there out of the range of the recording time (Gaja allosucked from Isis). These data further imply that allosuckling is common in the captive camels, comparable to captive guanaco [20], red deer [4], [9], cattle [10], and captive fallow deer [11], [12].

Up to three calves (always one filial and one or two non-filials, there were no more calves in the herd than three) were involved together in a sucking bout. The herd with the highest incidence of allonursing (25%) was the only herd with 3 calves providing the largest number of allonursing possibilities. The earliest allosucking was reported in 50-days-old calf. The youngest calves in the herd were never seen allosucking, despite of the fact that they had the possibility to do it just after joining the herd where other nursing female was present. In other ungulates, except in zebra [33], allosuckling was reported from the first day [4] or the first weeks [11], [20] of the calves' lives. Even calves with the large percentage extent of allosuckling (up to 100%, Table 2) allosucked only occasionally, being weaned by their mothers and using the opportunity to get surplus milk. Generally, the sucking bouts in this study were on average much shorter than those reported by Sambraus in dromedaries [30] (43 sec and 210 sec, respectively). We found tendency for longer duration of sucking bout for filial males than for filial females (similar to Paranhos da Costa *et al.* [13]). This may be caused by biased investment of females in good condition (with unlimited food supply in captivity) towards male offspring, as shown by Trivers & Willard [34] or simply by higher energetic demands by the larger sex. We did not find this difference for allosuckling bouts.

### Evidence for milk-theft hypothesis

Regarding the behaviour of calves, our results widely correspond with the milk-theft hypothesis. We confirmed that (i) allosucking calves sucked only in the lateral (other than antiparallel) position. That may have helped the calf to remain undetected by the nursing female or decreased the probability of being threatened by her. Higher incidence of allosuckling in lateral position was confirmed also by Zapata *et al.* [20] in guanacos. In contrast, the filial calves sucked mostly in the antiparallel position. There was no indication for changing the antiparallel to lateral positions with increasing age.

As predicted (ii), in all cases when more calves were sucking together, the non-filial calves joined filial calf during sucking non-maternal dam and the probability of allosuckling was higher when there were more calves involved in a sucking bout as reported by Ekvall [11], Zapata *et al.* [20] and Pluháček *et al.* [7]. In connection with the lateral position this reflects the obvious tactic not to be seen or threatened by the non-maternal nursing female, characteristic for the parasitisation for the surplus milk described by Packer *et al.* [1].

We failed to find any support for the prediction (iii). We did not record any case of non-filial calf sucking in anti-parallel position, so we could not assess any influence of sucking position on the termination by females. This could mean that calves which tried to allosuck close to the females head were not successful. Females might have refused to nurse them and calves then learned how to approach the non-maternal dam safely and successfully as reported by Zapata [20].

In agreement with our prediction (iv), the incidence of allosucking increased with age of the allosucking calf as skills of the calf to outwit the non-maternal dam increased or as increased the motivation of a calf due to the weaning process of weaning. At least one of the allosucking calves was already weaned. It corresponds with the findings of Ekvall [11] and Landette-Castillejos *et al.* [35], where the allosuckling occurrence increased with the length of lactation.

The suckling bouts generally lasted longer in filial calves in antiparallel position than in non-filial ones in lateral position as we expected (v). Although the sucking duration itself should not be used as a predictor of milk intake, it can reveal the level of maternal investment [36]. The sucking duration for non-filial calves which sucked alone was considerably shorter than in case when they joined already sucking filial calf. Sucking duration was longer for sucking bouts involving more than one calf. This may simply reflect the fact that in longer sucking bouts performed by a filial calf the non-filial calf got greater possibility to notice that the female is nursing, moved close to her and joined the sucking calf. The differences in sucking bout durations are also consistent with milk-theft hypothesis [1].

### Evidence for altruistic behaviour

On five occasions a non-filial calf was allosucking with no other calf present (ii). This could be simply a mistake from the dam, considering the fact that mentioned allosucking bouts were considerably shorter than those including also the filial calf. On the other hand, however, we cannot reject entirely the possibility that in some cases the dams tolerated certain individuals in need as an altruistic act as was reported for red deer [4], [9] and cattle [10]. Even when the non-filial calf sucked in the lateral position and together with filial calf, we cannot rule out the possibility that the females were able to recognize that they were nursing more than one calf at a time, because the size of allosuckling calves did not allow them to be completely hidden from the sight of the female even in the lateral position.

Termination of sucking bouts by the females did not differ during sucking events involving filial and non-filial calves. We could not test termination of a sucking bout involving a non-filial calf in an antiparallel position (iii) in comparison with a filial calf in the same position, because none of the non-filial calves has ever been seen in the antiparallel position. Taking into account that non-filial calves were sucking more often in the presence of the filial calf and that the non-filial calf was located more distant to the head of the dam, one could presume that the female would terminate equally sucking of filial and non-filial calves when trying to terminate the non-filial sucking. This may explain generally low level of terminations of non-filial sucking bouts and the tolerance of females.

The increasing incidence of allosucking in older calves (iv) may also imply higher tolerance of nursing females to calves that are more familiar to them as they had lived longer in the same herd than the newborn calves.

The fact that some dams allonursed while others did not, and the fact that at least some of the calves sucked regularly and very successfully suggests a possible strategy of compensation of nutritional requirements by the young as seen in red deer and cattle [9], [10]. Our data was not adequate for testing this possibility, however. On the other hand, body condition of females did not affect the probability of nursing non-filial calves in guanaco [37].

Age differences among calves in herds were larger in the zoos in this study (the first calf born in January while the last in July, see Tab. 2) than expected in the wild or in semi-captive conditions (several weeks in spring) [25]. This difference may be due to the prolonged breeding season in Europe, which may also increase the possibilities for allosuckling. Similar to Murphey *et al.* [38] and Cassinello [39] at the moment, we may exclude the kinship selection, as the females in the herd were not closely related to one another.

Our results correspond with those of Zapata *et al.* [20], [21] for captive and wild guanacos, where the ‘milk theft’ is most likely explanation of allosucking. As both camels and llamas are adapted to the extreme conditions, the allosuckling occurrence in captive animals could have two possible explanations. First, as camel females live probably in the kin groups [40], allosuckling could have developed as an adaptation for the harsh climatic conditions and can work on the principles of kin selection [1], [4], [9], [12] which should be the objective of further testing proposed also by Zapata *et al.* [20]. Second, females which are kept in less extreme conditions in captivity should have lost the care about what calf is sucking them, and the calves would exploit those possibilities. Moreover, the milk production of captive domesticated camels could be higher than the normal consumption of the calf because of the domestication changes and *ad libitum* food intake in females. Then the females may suffer from the milk overproduction, corresponding with the milk evacuation hypothesis postulated by Roulin [2].

## Conclusions

The results of the study support the hypothesis of ‘milk theft’, being mostly performed by calves behaving as opportunistic parasites. Nevertheless, tolerance of the camel females to non-filial calves may also suggest that at least in part allosuckling in camels might be adaptive trait, despite the fact it is mostly performed by calves which have the occasion to get surplus milk from a non-maternal female as opportunistic parasites.
